# Editorial: Assessment and treatment interventions for traumatic brain injury

**DOI:** 10.3389/fneur.2025.1696541

**Published:** 2025-09-29

**Authors:** Andrew P. Lavender, Ryusuke Takechi, Sarah C. Hellewell, Keisuke Kawata

**Affiliations:** ^1^Institute of Health and Wellbeing, Federation University Australia, Ballarat, VIC, Australia; ^2^Collaborative Evaluation and Research Centre, Federation University, Ballarat, VIC, Australia; ^3^Curtin Medical Research Institute, Curtin University, Bentley, WA, Australia; ^4^School of Population Health, Faculty of Health Sciences, Curtin University, Bentley, WA, Australia; ^5^Perron Institute for Neurological and Translational Sciences, Nedlands, WA, Australia; ^6^Centre for Neuromuscular and Neurological Disorders, University of Western Australia, Crawley, WA, Australia; ^7^Department of Kinesiology, Indiana University School of Public Health-Bloomington, Bloomington, IN, United States; ^8^Program in Neuroscience, The College of Arts and Sciences, Indiana University, Bloomington, IN, United States; ^9^Department of Pediatrics, Indiana University School of Medicine, Indianapolis, IN, United States

**Keywords:** brain injury, traumatic brain injury (TBI), brain injury assessment, mild traumatic brain injury (mTBI), chronic traumatic encephalopathy (CTE)

## Introduction

Traumatic brain injury (TBI) results from a blow to the head or other sudden head movement, often caused by collisions, falls, or impacts during sport, military service, or motor vehicle accidents.

Treatment options depend on severity, but mild TBI is frequently under-reported and often left untreated, particularly in sports where quick return-to-play is prioritized. Current assessment tools are limited in guiding safe recovery decisions, highlighting the need for improved evaluation methods. Rehabilitation strategies also require ongoing assessment and refinement to optimize recovery across the spectrum of injury severity.

This Research Topic invited contributions focused on assessment and intervention strategies for mild, moderate, and severe TBI. Twenty-one articles addressing TBI assessment and treatment in the form of original research, reviews, and a case report have been included.

## Preclinical insight: animal studies and mechanistic findings

Animal studies contribute greatly to critical mechanistic insights that inform development of interventions for rehabilitation following TBI. A study explored the long-term effects of decompressive craniectomy in TBI using a mouse model (Szczygielski et al.). Mice were divided into four groups: sham, craniectomy alone, TBI alone, and TBI with craniectomy. Neurological function and brain oedema were assessed over 28 days. Results showed that craniectomy following TBI worsened early neurological outcomes and increased brain oedema, transitioning from cytotoxic to vasogenic with gliosis. Craniectomy alone also caused delayed oedema and gliosis. Although the negative effects were transient, findings suggest that craniectomy may aggravate brain injury, and future treatments should aim to reduce post-surgical brain swelling to improve recovery.

TBI may disrupt thalamo-cortical pathways, triggering inflammation and vascular damage that contribute to neurodegeneration. Özen et al. explored the role of interleukin-1β (IL-1β) in capillary alterations and pericyte coverage in the thalamus and cortex following TBI. Using a central fluid percussion injury model in adult male C57BL/6 mice, researchers administered either a neutralizing anti-IL-1β antibody or a control antibody post-injury. Immunohistochemical analysis at 2 and 7 days revealed that early inhibition of IL-1β signaling prevented capillary damage and enhanced pericyte coverage in the thalamus, suggesting IL-1β plays a key role in post-TBI vascular pathology.

## Clinical prognostic tools and outcomes research

Network analysis was used in a study to examine symptom relationships in patients with mild traumatic brain injury (mTBI) treated at U.S. Level 1 trauma centers (Eagle et al.). Researchers analyzed Rivermead Post-Concussion Symptoms Questionnaire data from 1,593 patients at 2 weeks and 3, 6, and 12 months post-injury. Cognitive symptoms had the highest influence early on, while fatigue became most influential at 12 months. Emotional symptoms also showed significant influence, although network structure remained stable over time. Early symptom patterns were more predictive of long-term recovery and quality of life than traditional clinical factors, suggesting cognitive, emotional, and fatigue symptoms may be key targets for mTBI treatment.

A retrospective study by Generoso et al. examined risk factors for breakthrough early post-traumatic seizures (PTS) in patients with TBI receiving phenytoin prophylaxis. Among 105 patients in an intensive care unit (ICU), early PTS occurred more frequently in older individuals, those with higher Marshall Computed Tomography scores, and those undergoing neurosurgery for hematoma evacuation. Despite therapeutic phenytoin levels in most cases, seizures still occurred, and nearly half lacked level monitoring. Early PTS was associated with longer ICU stays and increased hospital mortality. These findings suggest that certain clinical factors may predict seizure risk despite prophylaxis, highlighting the need for improved monitoring and alternative strategies to prevent early PTS in TBI patients.

The study by Tang et al. included in this Research Topic developed and validated a nomogram to predict 28-day mortality in patients with skull fractures using data from the Medical Information Mart for Intensive Care (MIMIC) database with data from the eICU Collaborative Research Database used for external validation. A total of 1,527 adult patients were analyzed. Predictive factors included age, temperature, serum sodium, mechanical ventilation, vasoactive agents, mannitol use, extradural hematoma, loss of consciousness, and Glasgow Coma Scale (GCS) score. The model demonstrated strong performance, with AUCs of 0.857 and 0.853 and C-index values of 0.832 and 0.829 in training and validation sets, respectively. Calibration and decision curve analyses confirmed clinical utility, offering a reliable tool for early mortality risk assessment in skull fracture patients.

High sodium levels in TBI patients in ICUs are linked to poor prognosis, but research on optimal sodium levels and their impact on early mortality using the MIMID-IV database is limited. A retrospective study included in this Research Topic analyzed 1,749 TBI patients from the MIMIC-IV database to assess the impact of serum sodium levels on in-hospital mortality (Wang X. et al.). Patients with hypernatremia (sodium >145 mmol/L) had significantly higher mortality (25.3%) than those with lower levels (9.3%). A non-linear, L-shaped relationship was found, with mortality risk increasing sharply beyond 144.1 mmol/L. Even after adjusting for clinical variables, hypernatremia remained an independent predictor of death. These findings highlight the importance of monitoring and managing sodium levels in TBI patients, suggesting that targeted sodium control may improve outcomes and reduce early mortality.

TBI remains a major global health concern, particularly in low- and middle-income countries, where fatality rates are high despite existing guidelines. A key factor in severe TBI outcomes is elevated intracranial pressure (ICP), which can cause brain herniation and death. Timely diagnosis and management of ICP are critical in neuro-intensive care. Monitoring ICP offers real-time insights that help reduce secondary brain damage and improve survival. However, accurate interpretation still relies heavily on clinical expertise. This review article by Zhang et al. explores global disparities in TBI, the role and challenges of ICP monitoring, decompressive craniectomy, and the importance of personalized treatment approaches.

## Treatment/intervention trials

Systematic reviews provide particularly helpful insights into recent research. Kawata et al. evaluated randomized controlled trials on pharmacological, stimulation, and exercise-based interventions for chronic symptoms following TBI. Despite extensive research, pharmacological treatments—particularly neurostimulants like Modafinil and Armodafinil—showed inconsistent results in managing fatigue and sleep disturbances. In contrast, brain stimulation techniques (e.g., transcranial magnetic stimulation and blue light therapy) and exercise interventions demonstrated more consistent benefits for cognitive and mental health symptoms. However, most studies had small sample sizes, limiting their clinical impact. The review highlights the need for larger, high-quality trials to better understand and optimize treatment strategies for chronic TBI-related symptoms.

The next study in this Research Topic is a randomized, double-blind clinical trial that evaluated biperiden as a potential intervention to prevent post-traumatic epilepsy (PTE) in adults with acute TBI (Foresti et al.). Between 2018 and 2022, 123 participants were randomized to receive biperiden or placebo for 10 days, with outcomes assessed over 24 months. It is important to note that the trial was halted early due to the COVID-19 pandemic. Analysis showed no significant effect of biperiden on PTE incidence or mortality. Notably, the biperiden group experienced a higher frequency of late seizures. These findings suggest insufficient evidence for biperiden's efficacy in preventing PTE, highlighting the need for larger, more comprehensive studies on the use of biperiden as an intervention for PTE in adults with acute TBI.

A systematic review and meta-analysis evaluated the effects of amantadine on TBI recovery using data from six randomized controlled trials involving 426 patients (Félix et al.). Results showed that amantadine significantly improved GCS scores in patients than controls who received a placebo on day 7. Mini Mental State Examination Scores also improved indicating enhanced cognitive recovery. However, no significant differences were found in Disability Rating Scale, early GCS scores, Glasgow Outcome Scale, hospital stay length, or ventilation duration. While amantadine shows promise for short-term cognitive improvement in TBI patients, its broader impact on functional recovery remains uncertain and warrants further investigation.

The retrospective cohort study by Gao et al. investigated the impact of thiamine administration on in-hospital mortality among 1,755 TBI patients admitted to the ICU. Using data from the MIMIC-IV database, researchers found that thiamine use was significantly associated with reduced mortality, even after adjusting for confounding factors. Subgroup analysis revealed particularly strong benefits for patients aged 65 and older, males, and those with severe TBI. The findings suggest that thiamine may offer a protective effect in critical care settings for TBI patients, supporting its potential role in improving survival outcomes. Further research is needed to confirm these results.

## Functional and psychosocial outcomes

The study of Nicolau de Costa et al. assessed color discrimination in 10 cognitively normal TBI patients using a fixed saturation test and CT scans to locate brain lesions. All patients performed well with high saturation stimuli. However, four with ventral stream lesions and one with occipital damage showed impaired discrimination at low saturation. Those with dorsal stream lesions performed similarly to controls. The findings suggest that low saturation color discrimination deficits are linked to ventral brain damage and may serve as a functional tool for evaluating visual impairment in TBI patients.

Exertional tests have been suggested as a useful tool for assisting clinicians in the management of concussion. Some of these are expensive or require highly skilled clinical expertise for operation. The Multimodal Exertional Test (MET) was developed by Pyndiura et al. to assess concussion-related exertion using minimal resources. It includes four progressive stages combining physical and cognitive tasks. In a pilot study with 14 healthy interuniversity athletes, heart rate (HR) and symptom severity were monitored. HR increased from baseline to Stage 1 and again from Stage 3 to 4, with no significant changes between Stages 1 to 3. Female athletes showed higher HRs than males throughout. All participants reported minimal symptom changes. The MET effectively induced exertion without symptom provocation, suggesting its potential utility in concussion management with limited clinical resources.

mTBI can significantly impact patients' lives. Understanding the knowledge and attitudes of patients and their families is a crucial step toward enhancing patient care and management. The work of He et al. presents a cross-sectional study at Zhejiang Hospital in China that surveyed 573 individuals using an online questionnaire to assess mTBI knowledge and attitudes. Average knowledge and attitude scores were 11.00 (range: 0–20) and 27.78 (range: 8–40), respectively. Lower scores were associated with a middle school education and moderate-income levels, while personal experience with mTBI from falls correlated with more positive attitudes. These findings highlight gaps in awareness and the need for targeted, accessible education to improve understanding and attitudes toward mTBI, ultimately enhancing patient care and recovery outcomes.

TBI is a major cause of death and disability, especially in low- and middle-income countries (LMICs), where healthcare resources are limited. The original research study included in the Research Topic tested low-cost, scientifically validated tools—including paper-based and modified computer tasks—to identify post-TBI deficits in 51 patients and 20 controls across four Ethiopian hospitals (Semework et al.). Tests assessed cognitive and visual impairments, with the Montreal Cognitive Assessment (MoCA) proving especially effective. Results showed these methods can detect deficits in processing speed, memory, and executive function. The findings support the feasibility of implementing such tools in LMICs and highlight the need for dedicated rehabilitation centers.

The study by Wang J. et al. compared nurse-led and neurologist-led care for patients recovering from craniotomy due to TBI. Reviewing records of 230 patients over 6 months, researchers found that nurse-led care significantly improved activities of daily living, quality of life, and reduced anxiety, depression, and caregiver burden compared with neurologist-led care. Patients under nurse-led care also reported fewer complications, such as pressure sores and dizziness. These findings suggest that nurse-led follow-up care is not only effective but may surpass neurologist-led care for certain outcomes, highlighting its potential for improving long-term recovery and support in resource-limited settings.

Neuropsychological evaluation is the strongest predictor of functional outcomes in TBI patients. While research on TBI diagnosis and treatment has grown rapidly, there is a lack of comprehensive, practical neuropsychological reviews. Chan et al. provide another review that expands beyond standard overviews by examining the rationale behind neuroimaging choices and recent findings on neuropsychological assessments and their link to recovery. It includes tables detailing recovery trajectories across age groups, associated risk factors, and promising cognitive rehabilitation tools. Phenomenological studies are also discussed. The review concludes by identifying research gaps and suggesting future directions to enhance clinical understanding and treatment of TBI.

## Case reports and rare conditions

Chronic subdural hematoma (CSDH) is typically linked to trauma but rarely associated with nephrotic syndrome. This case report describes a patient with nephrotic syndrome who developed CSDH (Xue, Xue et al.). Laboratory tests revealed hypoproteinemia and hyperlipidemia, confirming nephrotic syndrome. The patient underwent successful trepanation and drainage of the hematoma, followed by atorvastatin treatment. After neurological improvement, the patient was transferred for nephrotic syndrome management. No neurological issues were observed at a 3-month follow-up. The case suggests nephrotic syndrome may contribute to CSDH and highlights the importance of timely surgical intervention and coordinated care for optimal recovery.

The next article describes a retrospective study comparing clinical characteristics of traumatic and not otherwise specified (NOS) chronic CSDH in 301 patients (Yang et al.). Traumatic CSDH patients were generally younger, more likely to experience seizures, and less likely to be on anticoagulant or antiplatelet medications. They also had a higher rate of asymptomatic presentation. Despite these differences, both groups showed similar radiological findings at admission, received comparable treatments, and had equivalent clinical outcomes. The study highlights distinct patient profiles for traumatic vs. NOS CSDH but suggests that treatment approaches and prognoses remain consistent across both types.

Penetrating brain injuries are rare but highly lethal and disabling forms of neurosurgical trauma. Skull base injuries caused by arrows are especially uncommon and typically accidental, though self-inflicted cases are rising. This report presents a unique case of a suicidal transoral arrow injury penetrating the brain (Xue, Li et al.). It emphasizes the importance of preoperative imaging for surgical planning and confirms surgery as an effective treatment. The study also reviews previous case reports to offer clinical recommendations for detecting and managing such injuries, highlighting the need for careful assessment and tailored intervention strategies in these complex and life-threatening cases.

An opinion article by Omer et al. discussed a study on management and treatment of patients with traumatic tension pneumocephalus and the challenges of treating people with brain injury in low- and middle-income countries (1). The authors clearly describe the condition, explaining that it is a compressive effect caused by air trapped on the brain, and recent studies that have explored possible techniques for resolving the condition. The authors suggest that the priority should be to reduce hospital stays and risk of post-operative complications and that this is most likely achieved using endonasal endoscopic approaches for first-line treatment. They argue that this method is preferred since it is the least invasive approach, prioritizes efficiency and safety, and is accessible to people in low- and middle-income countries.

Together, this collection of articles adds significant knowledge and advances our understanding of various aspects of assessment and treatment of TBI using human and animal original studies as well as reviews, case reports, and an interesting opinion article.
